# Reviews and Book Notices

**Published:** 1883-10

**Authors:** 


### Reviews and Book Notices.

ARTICLE XII.—THE GYNÆCOLOGY OF THE ANCIENTS. By EDWARD W. JENKS, M.D., of Chicago, Ill. Translated from the English by PROF. DR. LUDWIG KLEINWACHTER.

The leading gynæcologists of America came together in the year 1876 and resolved to form a gynæcological association, whose workings might extend throughout the entire country.

As is usual in North America, the conception is followed by speedy perfection.

The association was organized, and it was resolved that annual meetings should be held in one of the larger cities and its proceedings published in a yearly volume known as the Transactions of the American Gynæcological Association. Six of these volumes have already appeared. They are an ornament to our professional library, interesting in the highest degree, and containing such valuable writings as might be expected from the coöperation of such men as Washington Lemuel Attlee, Fordyce Barker, Nathan Bozemann, William H. Byford, James Chadwick, Thomas Addis Emmet, Geo. Engelman, William Goodall, Charles Carroll Lee, William Lusk, Paul Mundé, Emil Nöggerath, Theophilus Parvin, Randolph Peaslee, and the renowned J. Marion Sims, T. Gaillard Thomas and others whose literary names are everywhere known.

Aside from the style and manner of the literary handling of matter (in which every nation differs), one encounters an essential difference between the Transactions and our similar literary productions. In this, each volume, up to date, contains as a leader a medical history or geographical work.

The earlier volumes give a full biography of Simon (of Heidelberg), Charles Buckingham, Randolph Peaslee, Marmaduke B. Wright, and others.

The fifth volume contains Engelmann's great work, with many neat illustrations, upon Aid During Chidbirth, by different writers of our own continent, and the following, sixth, a very noteworthy production from the pen of Edward W. Jenks, of Chicago, Ill., entitled "The Practice of Gynæcology in Ancient Times."

Here, as in many other respects, are we humiliated by the celebrated hyper-realistic North American.

With us the literature of the day, with one exception—namely, Virchow's Archives—holds aloof from any history of medicine, as well as medical geography; indeed, are often directly antagonists of same. We have, then, to thank principally the worthy manager of this journal that we cannot even now be made, in this matter, the reproach of other nations. We did neglect the history of medicine entirely.

We would not be so surprised, if the English or French cultivated the history of medicine, for these two nations possess more than a thousand years of glorious history, and one of such a nature as to inspire historical research.

But the North American is deficient in a national ancient culture, and likewise in ancient history, and in spite of this we se them encouraging and advancing the history of medicine. I consider this an indication worthy of notice. It is clear that these people are possessed with a desire to know what the ancients knew and did in order not to become bigoted—in other words, to seize completely upon the true spirit of medicine, whereby the so-called exact research became possible.

---

ARTICLE XIII.—A TREATISE ON THE MATERIA MEDICA AND THERAPEUTICS OF THE SKIN. By HENRY G. PIFFARD, A.M., M.D., Professor of Dermatology, Medical Department, University of the City of New York. 800, cloth, pages 851. Wood's Library of Standard Medical Authors, Feb., 1881.

This book possesses the advantage of an American authorship and will escape the appellation of literary theft, which applies to a large proportion of the cheap medical works published now-a-days. The first part (pp. 117) contain the drugs, which are of use in the treatment of skin diseases, arranged in alphabetical order, and very methodically. First is given the effect of a drug on the integument when taken internally, then when applied locally, and thirdly, the diseases in which its use has been recommended, with reference to the page where such diseases are treated of in the second part. About 414 drugs are mentioned in the first part.

Part II. is devoted to the Therapeutics of Skin Diseases. After giving a short description of the disease and its etiology, the author enters into its methodical treatment at length, and gives in an appendix at the end of each article a list of all the medicines used in that disease, and references to part I.

A bibliography of 210 authors is contained in this volume, together with a formulary of over a hundred and forty prescriptions, all of which are expressed in the metric as well as in the old systems.

The favor with which Dr. Piffard looks upon homœopathy is traceable in his book, where he recommends in eczema the hundredth part of a drop of the tr. of *Rhus Toxicodendron.*

However, we are of the opinion, that although this treatise is inferior to the classical works of Duhring or of Hyde, yet it has an aim of its own in calling the attention of the readers to treatment, and will give the busy practitioner more information than a number of compendia issued within a few years past for the purpose of ready reference. H. D. V.

---

ARTICLE XIV.—LEGAL MEDICINE. By CHARLES MEYNOTT TIDY., M.B., F.C.S., Master of Surgery, Professor of Chemistry and of Forensic Medicine and Public Health at the London Hospital; late Deputy Medical Officer of Health, and Public Analyst for the City of London, etc., etc.

Volume I.—Evidence; The Signs of Death; Identity; The Cause of Death; the Post-Mortem; Sex; Monstrosities; Hermaphrodism.

Volume II.—Expectation of Life; Presumption of Death and Survivorship; Heat and Cold; Burns and Scalds; Lightning; Explosives and Combustibles; Starvation, its Treatment. 8vo., cloth, pp. XXII–314 and XII–298. November and December issues of Wood's Library of Standard Medical Authors for 1882.

It is impossible to give more than an opinion of the value of this work, the third (and last?) volume being announced for another year's issue. This will appear more or less an abuse to some subscribers, and while it may forcibly retain some old ones, it may be cause that somebody will be deterred from subscribing to the series of 1883, which is thereby linked to that of the precedent year.

In the present volumes, medical jurisprudence has received an amplification which the intricacies of the law, as practiced in our days, necessitated. The work of the author is methodical, and, strange to say, his style is highly interesting, in spite of the dreariness of the subject. It would also appear that the basis of these lectures was laid by a colleague prematurely lost to the profession. However that may be, no one would look upon these volumes as a mere compilation, still less as the outgrowth of a few years of study, and it is very likely that they will become standards of medical jurisprudence. No one will regret his subscription to the library on account of these volumes being thrown in. H. D. V.

---

ARTICLE XV.—A TREATISE ON THE PRACTICE OF MEDICINE, FOR THE USE OF STUDENTS AND PRACTITIONERS. By ROBERT BARTHOLOW, M.A., M.D., L.L.D. Third edition, revised and enlarged, 800, cloth, pp. xx–918, New York; D. Appleton & Co., 1882.

The Ideal Practice. Bartholow has accomplished in this work a result of which the whole profession had despaired. Decade after decade, editions after editions of antiquated works had rained on the profession of medicine in the United States, the treatment advocated in which would make a student smile. After a while it was thought that a translation of a standard foreign work would supply something new, and it did: more infusions and powdered drugs and teas than our western drug-stores contain found their way through Ziemssen's Cylopædia into the students brains. It was a great relief to find that Prof. Bartholow possessed good common sense besides erudition, but for this very reason, his work is above criticism. It is one of those treatises the happy possessor of which would wish that he had a patent on, so that none of his neighbors could read it and learn. It is no wonder to us that Bartholow's practice has been left out of several medical colleges' catalogues; most likely its introduction in them would oblige some old professors to take many lessons of therapeutics as applied in our days. But the rapidity with which editions are exhausted is a surprise to no one acquainted with the book. H. D. V

---

ARTICLE XVI.—THE ESSENTIALS OF PATHOLOGY. BY PROF. D. TOD GILLIAM, of Starling Medical College, Columbus, O. Philadelphia: P. Blakiston, Son & Co. 1883. P. 296.

In this busy era of investigation, discovery and discussion, in the ever widening fields of medical science, the immensity of the accumulation tends to discourage and confuse the young student. It is but fitting, then, that the most essential features of the science of pathology should be presented by one familiar with them, in plain, practical style—not to completely acquaint the student with the subject, but to bring its salient points within easy comprehension, increasing thus his interest in the subject. Hence the author of this work avoids in great measure the discussion of unsettled subjects, for the sake of preventing confusion in the mind of the student, and rendering more clear and distinct his conception of the generally accepted doctrines of to-day. There are thirty-three chapters in the book, treating briefly and clearly of Normal Histology, of Constructive and Destructive Processes in Disease; of Degeneration, Infiltrations, Metamorphoses, Death, Mechanical and Functional Derangements, Fever, Tumors, New Formations, etc.; with chapters on the pathology of the blood, and each of the different tissues of the human body. The general practitioner will also find in this little 12mo. a convenient compendium of the current pathology of the day.

---

ARTICLE XVII.—COULSON ON THE DISEASES OF THE BLADDER AND PROSTATE GLAND. Sixth edition. Revised by WALTER J. COULSON, F.R.C.S., Surgeon to St. Peter's Hospital for Stone, etc., and Surgeon to the Lock Hospital. 8vo, cloth, pp. XXIV—393. New York: Wm. Wood & Co. 1881. July issue of Wood's Library of Standard Medical Authors.

Few authors have treated the subject to such an extent, and this book is cyclopædic in character. All the prominent writers are passed in review, their opinions analyzed, and their treatment epitomized. Litholopaxy is treated of in a chapter by itself, and is highly recommended. The surgical treatment of the various diseases is commendable throughout, but the medication advocated well represents the standing of the profession in Europe, and is not at all adapted for our more refined mode of prescribing. Such things are advocated as a large number of infusions, crude drugs, pure balsams, etc., which few patients could stomach, and very seldom is a prescription written out as it should be. This book is illustrated, printed in a clear type, and specially valuable for surgeons and practitioners who make a specialty of genito-urinary diseases. H. D. V.

---

ARTICLE XVIII.—OCTAVO SERIES, STANDARD MEDICAL BOOKS: THE DISEASES OF WOMEN. By GRAILY HEWITT, M.D., F.R.C.P. Fourth American, from the last revised and enlarged London Edition. With 132 illustrations. Philadelphia: P. Blakiston, Son & Co. 1882. Two parts in one vol., $1.50. Pp. 751.

"No subscribing nuisance is connected with this series; each volume is sold separately." This is a sample of the animosity which has sprung up between half a dozen medical publishers in this country since the Wood Library was started. The practice of issuing paper covers is a rare thing in the medical literature of America, but it is quite common in France, and seems to naturally favor the poorer class of the profession. In the present case, for instance, a most valuable manual is placed in the hands of students at a nominal cost. THE JOURNAL AND EXAMINER had occasion once before to review very favorably this same work. Hardly any more can be said than that it is one of the best works ever written on the Diseases of Women, and answers the purpose of any other. The same can be had bound in cloth for $2.50. The printing, the paper, and the illustrations are of the best quality. H. D. V.

---

INTERMITTENT FEVER TREATED WITH ELECTRICITY.

Electricity has been used by Frank, Borgini, Aldini and others; in these later times by Bossi, of Rome; by Vizioli, of Naples; by Shipulski, Krasnogladof, Deparquet, etc. Prof. DeRenzi, of Genoa, has also largely experimented with it, and has found that in the majority of cases, the fever is stopped, and frequently more promptly than with quinine. In nine cases, the author has had five complete cures, two bettering, and two with no success. They were treated with the continued and the faradaic current; the first obtained with 9 to 62 elements, and applied five to fifteen minutes along the spinal cord. The faradaic current has been more efficient than the galvanic. These experiments have confirmed the possibility of conquering intermittent fever with electricity; but so far, it has been impossible to ascertain why in some cases a rapid and complete cure is obtained, and in others an incomplete one, and what are the best means of application of electricity, and when it ought to be preferred to quinine.—*Annals Univerzali.*

THE interesting and valuable article on the Use of Lactic Acid in Diphtheria, by Dr. Lytle, was so dreadfully mangled by the printers that we republish it, and hope our readers will excuse the blunders we made in the former proof-reading.

